# A LONGITUDINAL ANALYSIS OF EVERYDAY DISCRIMINATION

**DOI:** 10.1093/geroni/igae098.1369

**Published:** 2024-12-31

**Authors:** Felicia Wheaton

**Affiliations:** Xavier University of Louisiana, New Orleans, Louisiana, United States

## Abstract

Experiences of everyday discrimination are associated with poorer mental and physical health in later life. However, most studies examine discrimination at a single time point. There is a need to better understand how experiences of everyday discrimination change over time, change with age, and differ by cohort. This study examined longitudinal change in everyday discrimination using data from the 2006-2020 waves of the Health and Retirement Study (HRS), a nationally-representative study of Americans 50+ (N=23,523). Frequency of everyday discrimination was summed across 5 items: you are treated with less courtesy or respect than other people, receive poorer service than other people at restaurants or stores, are threatened or harassed, people act as if the think you are not smart, and act as if they are afraid of you (1=never, 6=almost every day). Overall, everyday discrimination discrimination decreased from 2006 until 2016 when it peaked again, then fell through 2020. Average everyday discrimination increased across cohorts, with the oldest cohort (born before 1924) having the lowest average discrimination and the late Baby Boomers (born 1960-1965) experiencing the highest average discrimination. Across waves, as people aged, their discrimination scores decreased. The adjusted linear mixed model confirmed these trends: age was negatively associated with everyday discrimination and younger cohorts reported higher everyday discrimination. These findings indicate that age, period and cohort differences exist in experiences of everyday discrimination, and this has important implications for health across the life course.

